# The quintessence of algal biomass in bioplastic production: insightful advancement and sustainable use

**DOI:** 10.1186/s40643-025-00908-2

**Published:** 2025-08-25

**Authors:** Khushboo Iqbal, Arti Mishra, Smitha Mony Sreedharan

**Affiliations:** 1 Amity Institute of Microbial Technology, Amity University, Noida, 201313 India; 2 Department of Botany, Hansraj College, University of Delhi, Delhi, 110007 India; 3 Umeå Plant Science Centre (UPSC), Department of Plant Physiology, Umeå University, 901 87 Umeå, Sweden

**Keywords:** Algal bioplastics, Algal biorefinery, Polyhydroxyalkanoates (PHA), Genetic engineering, Life cycle assessment (LCA), Circular economy

## Abstract

Plastics are essential components of modern life, and their global demand is increasing daily. They are gaining recognition as a sustainable source for bioplastic production due to their rapid growth, carbon fixation ability, and capacity to utilize various waste streams. It seems that landfill, incineration, chemical treatment, and plastic recycling are not the best options for minimizing plastic pollution. A novel approach A new approach is needed to reduce this pollution. Bioplastics are biodegradable and come with less toxicity, a low carbon footprint, and are a better alternative to fossil-based plastics. This review explores recent advances in algal bioplastics, focusing on key polymers like polyhydroxyalkanoates (PHAs) and polylactic acid (PLA). Special attention is given to the use of genetic tools such as CRISPR-Cas systems to improve yield and carbon flux. Challenges related to downstream processing, low biomass productivity, and environmental variability are also discussed. This review highlights the importance of standardized life cycle assessments (LCAs) to evaluate environmental impact across the entire production chain. Additionally, regulatory frameworks from different countries are compared to identify gaps and promote progressive policy development. The review aims to provide an integrated perspective on the technical innovation, economic feasibility, and policy needed to support the future of algae-based bioplastics.

**Graphical Abstract:**

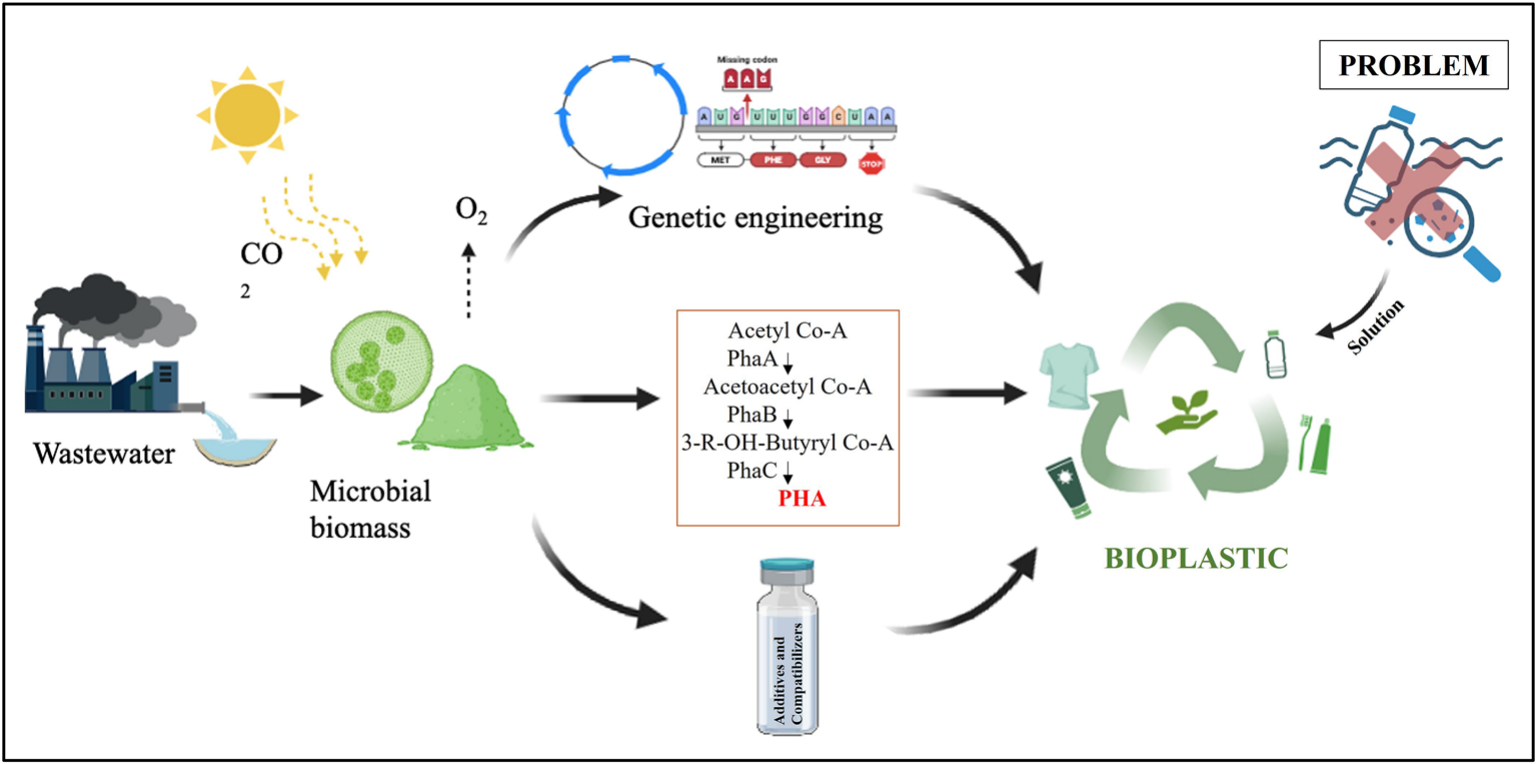

## Introduction

The escalating global consumption of plastic products has precipitated a crisis of unprecedented proportions, characterized by the accumulation of persistent plastic waste and its harmful effects on ecosystems worldwide (Rahman et al. 2017). Over the past few decades, the surge in plastic production, fuelled by its versatility, durability, and low cost, has led to alarming pollution levels across terrestrial, aquatic, and even atmospheric environments. In recent years, more than 368 million metric tonnes of plastic-based materials have been produced globally (Rajpoot et al. 2022). Approximately 8 million tonnes of plastic debris are discharged into the marine environment each year. This poses a significant threat to aquatic biodiversity and food webs (Cywar et al. 2022). The European Commission has reported that roughly 4% of global plastic production ends up in the oceans annually, underscoring the urgent need for effective waste management strategies and sustainable alternatives. In Europe, despite ongoing efforts to promote recycling, only 30% of the 25.8 million tons of plastic waste generated each year is recycled, leaving a substantial portion to be incinerated, landfilled, or released into the environment (Arora et al. 2023). A report further highlighted that only 25% of plastics were recycled, with 22% subjected to incineration, and the remainder ending up in landfills, where they can persist for centuries, leaching harmful chemicals into the surrounding soil and water resources (Arora et al. 2023).

The environmental consequences of this ubiquitous plastic pollution are far-reaching and multifaceted. The accumulation of plastic waste in landfills not only consumes valuable land resources but also contributes to soil and water contamination (North and Halden 2013). Marine plastic debris poses a direct threat to marine life through entanglement, ingestion, and habitat destruction (Thiruchelvi et al. 2021). Microplastics, resulting from the fragmentation of larger plastic items have been found in various marine organisms, including commercially important fish species, raising concerns about the potential transfer of harmful chemicals and pollutants to human consumers (Cywar et al. 2022). Moreover, the incineration of plastic waste releases greenhouse gases and other air pollutants, exacerbating climate change and respiratory health problems (Arora et al. 2023). The COVID-19 pandemic further amplified the crisis with increased demand for single-use plastics in healthcare and other sectors. It has been estimated that over 1.6 million tonnes of plastic waste were generated daily during peak pandemic periods (Benson et al. 2021). Traditional fossil fuel-based plastics are inherently non-biodegradable, often persisting for centuries. As awareness of their long-term impact has grown, attention has turned towards sustainable alternatives, including bioplastics. The term"bioplastics"is often used ambiguously and loosely and can encompass a wide range of materials with varying properties and environmental impacts. In its broadest sense,"bioplastic"refers to any plastic material derived from biological sources, such as plants, microorganisms, or agricultural waste. Biobased plastics are derived from renewable biological sources (e.g., starch, sugar), whereas biodegradable plastics can break down through microbial action into water, carbon dioxide, and biomass. However, not all bioplastics are biodegradable, and not all biodegradable plastics are compostable under natural conditions. Compostable plastics, a subset of biodegradable plastics, decompose under specific industrial or home composting conditions, leaving no toxic residues. This distinction is vital for life cycle assessment (LCA) and policy development. Bioplastics, if correctly defined and categorized, can play a pivotal role in reducing fossil resource dependency, mitigating climate change impacts, and promoting circular economic models. Among various sources of bioplastics, algae comprising both microalgae and macroalgae have emerged as a promising feedstock. Their rapid growth rates, high carbon fixation efficiency, and ability to grow on non-arable land or in wastewater environments make them attractive from ecological and economic perspectives standpoint. Moreover, algal biomass can produce valuable precursors such as carbohydrates, lipids, and proteins that are suitable for bioplastic synthesis (Sudhakar et al. 2024). Recent developments have demonstrated the feasibility of converting algal biomass into biodegradable polymers such as polyhydroxyalkanoates (PHAs), polylactic acid (PLA), starch-based plastics, and bio-based polyethylene (PE) (Mishra et al. 2021). PHAs are biocompatible polyesters produced by microorganisms, including cyanobacteria and engineered algae, as intracellular carbon and energy storage (Mogany et al. 2024; Arora et al.^2023^). For instance, *Ulva* sp., a marine green alga, can be used to generate biodegradable bioplastics using a simplified method that utilizes the whole algal biomass (El Semary et al.^2022^). PLA is derived from fermented sugars and exhibits thermoplasticity, making it suitable for packaging and biomedical use. Starch-based plastics, though cost-effective, require blending with other biodegradable polymers to improve mechanical properties (Naser et al. 2021). Bio-based polyethylene is chemically identical to conventional PE but is derived from renewable sources such as sugarcane or vegetable oils, offering a direct substitute using renewable carbon inputs (Degli et al. 2021).

Despite the advantages of biodegradable bioplastics, significant challenges still remain. Algal cultivation and downstream processing-harvesting, cell disruption, and polymer extraction are often energy-intensive and costly (Mogany et al. 2024). Additionally, yield variability due to environmental fluctuations and strain-specific growth dynamics can impact the consistency of biopolymer production (Sharma et al. 2024a; b). Advancements in genetic engineering offer potential solutions. Tools such as CRISPR- Cas systems are being used to optimize metabolic pathways in algal strains, enhancing carbon flux towards target polymers (Wang et al. 2023). Engineered algae can be tailored for higher biopolymer content, tolerance to environmental stress, and utilization of diverse substrates. However, these modifications must be balanced against biosafety concerns and regulatory scrutiny, especially when deploying genetically modified strains in open systems (Kumar et al. 2015; Ugya et al. 2024).

This review aims to provide a comprehensive and critical assessment of the current state of knowledge regarding the use of algal biomass in bioplastic production. It explores the types of polymers derived from algae, the underlying production technologies, and the role of genetic tools in enhancing yields. It also evaluates the challenges of downstream processing, environmental variability, and the need for comprehensive regulatory frameworks. It emphasizes the integration of bioplastic into a circular economy, supported by sustainable life cycle strategies and progressive policy models across global jurisdictions.

## Biopolymers produced from algal biomass

Biopolymers are naturally occurring polymers produced by the cells of living organisms. These biopolymers are composed of monomeric units that are covalently linked to form larger molecules (Roy et al.^2024^). These large biological molecules consist of three classes, categorized according to the monomers used and the structure to form a larger molecule: (i) Polynucleotides: These include RNA and DNA, consisting of 13 or more nucleotide monomers. (ii) Polypeptides: Proteins that are composed of over 100 amino acid monomers (example, collagen, actin, fibrin, etc.). (iii) Polysaccharides: Long chains of monosaccharide units combine to form polymers called polysaccharides (Jaiswal et al.^2019^). A distinct polysaccharide with a particular structure and composition is created depending on the monosaccharides present along the chain and the type of bond involved. They are the polymeric carbohydrates which can be linear or branched, such as starch, glycogen, alginate, etc. (Jaiswal et al. 2019).

Algae abundant in diverse ecosystems serve as a promising and sustainable feedstock for producing biopolymers. They are capable of growing on non-arable land, utilizing wastewater and achieving high photosynthetic efficiency, making them cost-effective and eco-friendly (Khoo et al. 2019; Lutzu et al. 2021; Parsons et al. 2020). Recent studies emphasize the integration of algal biomass in biorefineries for the co-production of bioplastics, biofuels and other high-value products, enhancing overall economic viability (Maheshwari et al. 2021; Dutta et al. 2025). However, scalability and economic viability remain major challenges. Producing algal biopolymers on an industrial scale requires significant advancement in genetic engineering, cultivation technique and downstream processing to reduce cost and enhance yield (Samoraj et al.^2024^).

### Polyhydroxyalkanoates (PHAs)

PHAs are biodegradable aliphatic polyesters synthesised by various microorganisms, including algae or bacterial cells as an intracellular storage compound for carbon and energy (Madadi et al.^2021^). Although their manufacturing on a broad scale is still in its infancy. These environmentally benign biopolymers offer significant potential in the domestic, agricultural, industrial, and biomedical fields. They originate from a variety of sources, including wastewater, glycerol, industrial wastes, agricultural waste, domestic waste, and lignocellulosic raw materials, and are formed by various microbes (Du et al. 2012). PHAs are typically accumulated in cells as granules encased in a phospholipid-protein membrane, enabling microorganisms to survive under nutrient stress. PHAs are known to contain about 150 distinct hydroxy alkanoic acids, and it has been reported that microbes from over 90 genera acquire these polyesters (Castilho et al. 2009). The hydroxy acid monomers are often 3-hydroxyalkanoates (3-HAs), 4-hydroxyalkanoates (4-HAs), or 5- 5-hydroxyalkanoates, with saturated and unsaturated chains that contain or do not contain aliphatic and aromatic groups (Albuquerque et al. 2018). PHA synthesis is influenced by the chemical composition of the carbon substrate added to the growth media as well as the metabolic capabilities of the microalgae. Cyanobacteria are frequently researched for the strategies to enhance synthesis under conditions of limited nitrogen or through genetic engineering investigations (Sreenikethanam and Bajhaiya et al. 2021). The absence of nitrogen can induce stress, triggering the production of biopolymers. *Synechococcus subsalsus* and *Spirulina* have been used in the development of the novel 14–18 carbon chain PHA biopolymer, whereas *Chlorella minutissima* did not exhibit PHA polymer production even under nitrogen deficiency (Kartik et al. 2021). In the form of hydroxyacyl-CoA, the microorganisms are capable of producing intermediates at different stages of the metabolism, which is the main enzyme for PHA production, and is identified and polymerized by the PHA synthase enzyme (Silva et al. 2007). PHAs can be categorised into three classes on the basis of the monomer size: short chain lengths monomer (SCLs) where the residues contain 3–5 carbon atoms, medium chain lengths (MCLs) generate residues with 6–15 carbon atoms, while long chain lengths (LCLs) contain residues with more than 15 carbon atoms (Simon-Colin 2012). PHA synthase is substrate-specific; the difference in the chain lengths can be seen. A specified range of 3-hydroxyalkanoic acids with different carbon lengths can be administered to PHA synthase. Recent studies have demonstrated significant improvement in PHA production using algal biomass. The PHA yield from algae often varies based on the cultivation conditions and strain specificity. For instance, *Chlorella pyrenoidosa* (27%) (Devadas et al. 2021), *Synechococcus subsalsus* (16%) and *Spirulina* sp. (12%) (Costa et al. 2020), *Phaeodactylum tricornutum* (10.6%) providing insights into PHAs content under similar conditions. Guo et al. (2024) reported that genetically engineered strains of chlorella exhibited enhanced PHA yields through the integration of lignocellulose fraction enhancement techniques (Guo et al. 2024). Furthermore, the use of waste glycerol as substrate has reduced production cost, making the process economically viable as compared to bacteria (Elsayed et al. 2024). In comparison, over 300 different microorganisms have been explored or producing biopolymers. However, only a few bacterial strains bacterial strains are widely used for biopolymers due to their high efficiency. These include *Cupriavidus necator* (Kusuma et al. 2024), *Ralstonia eutropha* (Di Stadio et al. 2024), *Bacillus megaterium*, *Bacillus cereus*, *Bacillus Subtilis, Pseudomonas aeruginosa*. These strains are preferred for their strong production rate and reliability and often achieve PHA yields exceeding 80–90% of cell dry weight when cultivated under optimized conditions (Koller et al. 2017). Out of these, *Bacillus megaterium* is highly efficient for producing PHA due to its robust gene activity. Additionally, its lipopolysaccharide-deficient cell wall structure enhances the recovery of intracellularly accumulated PHAs (Cal et al. 2021; Martínez et al. 2024). These systems can convert various carbon substrates, including glucose, glycerol, molasses and volatile fatty acids, into PHAs with high efficiency. However, such processes typically require sterile fermentation conditions, precise pH and aeration control and high-purity feedstocks, which can inflate operational cost and limit scalability (Koller and Obruča 2021) (Table 1). Recent advances have sought to mitigate these limitations through the use of waste-derived substrates (e.g., crude glycerol, fruit peel, and food processing waste), enabling cost-effective and sustainable PHA production in non-sterile setups (Rivas-Castillo et al. 2024). For instance, marine bacteria such as *Halomonas campaniensis* LS21 can grow in non-sterile conditions using mixed substrates (Yue et al. 2014), while isolates like LAMA 677 and LAMA 685 effectively utilize glycerol for PHA production (Takahashi et al. 2017).

**Table 1:** Comparison of PHA production across different microbial strains

Strain	Type	Substrate	PHA Yield (% DCW)	Method/Condition	Scalability Challenges	References
*Chlorella pyrenoidosa*	Microalga	Glucose	27%	N-limitation + batch cultivation	Moderate yield, sensitive to contamination	Devadas et al. (2021)
*Spirulina sp.*	Cyanobacteria	Waste glycerol	12–22%	Salinity stress	Moderate scalability, requires pH control	Costa et al. (2020); Mourão et al. (2020)
*Phaeodactylum tricornutum*	Microalga	Sulfur-deprived media	10.6%	Photoperiod 14:10	Low productivity, expensive photobioreactors	Hempel et al. (2011)
*Cupriavidus necator*	Bacterium	Fructose, CO₂	~ 90%	Fed-batch fermentation	High yield, high substrate cost	Bellini et al. (2022)
*Halomonas campaniensis*	Marine Bacterium	Mixed fatty acids	60–70%	Non-sterile, saline environment	Needs halophilic reactor conditions	Yue et al. (2014)
*Bacillus megaterium* MNSH1-9 K-1	Bacterium	Fruit peel hydrolysates	52%	Open culture, enzymatic pretreatment	Variable carbon content in feedstock	Rivas-Castillo et al. (2024)
*Chlorella fusca*	Microalga	Pentose sugar	High (exact % not defined)	Static culture + gene upregulation	Slow growth, PHB recovery difficulty	Cassuriaga et al. (2018)
*Pseudomonas putida* KT2440	Bacterium	Fatty acids, glycerol	~ 65%	Accumulation reactor	Requires multi-stage processing	Le et al. (2012)
*Synechococcus subsalsus*	Cyanobacteria	Glucose under N-deficiency	16%	Controlled photobioreactor	Genetic tools still under development	Kartik et al. (2021)

Algal biomass presents a compelling alternative to traditional bioplastic feedstocks, offering a unique balance of ecological and productivity advantages. Unlike bacterial fermentation feedstocks (e.g., sugars from corn or sugarcane), algae do not require freshwater or fertile land, making them particularly attractive in water-scarce or marginal environments (Kaloudas et al. 2021). Compared to lignocellulosic biomass (e.g., crop residues, wood), algae exhibit significantly higher productivity per unit area due to their rapid growth rates and ability to thrive in saline or wastewater, thereby reducing land-use pressure and avoiding competition with food crops (Kaloudas et al. 2021). Moreover, algal systems contribute to environmental remediation and enhanced carbon fixation, positioning them as a long-term sustainable feedstock for bioplastic production (Sharma et al. 2024a; b). However, algal cultivation and biomass harvesting remain energy-intensive, especially in closed photobioreactor systems, and the extraction of bioplastics or precursors (like PHAs or starches) from algal cells is still costly and technically complex (Osman et al. 2023). In contrast, lignocellulosic feedstocks are abundant and inexpensive but are hampered by challenging processing requirements, such as the need for harsh pretreatment to break down cellulose and lignin, which increases energy demand and cost (Abolore et al. 2024). Bacterial systems, while offering the highest polymer productivity per unit biomass and high product specificity, are constrained by the high cost of refined substrates and the complexity of controlled fermentation processes. Thus, while each feedstock presents trade-offs, algal biomass offers a uniquely integrated solution—combining moderate polymer productivity with low land and water use, environmental benefits, and carbon capture potential—making it a promising candidate for sustainable bioplastic production (Table 2).

**Table 2:** comparative assessment of Biopolymer feedstock based on sustainability and productivity and limitations

Feedstock	Biopolymer Types	Land/Water Use	Productivity (Yield %)	CO₂ Sequestration	Energy Demand	Cost Efficiency	Key Limitations	References
Algal Biomass	PHA, PLA, Starch, Alginates, Cellulose	Non-arable, saline/wastewater	10–35% DCW; up to 4 g/L/day (*Chlorella*, *Spirulina*)	High	Moderate (drying, harvesting)	Improving with biorefineries	High moisture content; scale-up challenges	Mohan et al. (2022); Vickram et al. (2023)
Lignocellulosic Waste	PLA, PHA (via bacterial processing)	High; forestry and crop residues	10–40% (after pretreatment)	Low	High (due to recalcitrance)	Low but feedstock is abundant	Expensive pretreatment; lignin interference	Goswami et al. (2022)
Bacterial Fermentation	PHA (e.g., PHB, PHV), PLA	Indirect (sugar/starch-based feed)	Up to 90% CDW (*Cupriavidus necator*, *Ralstonia eutropha*)	Low	High (sterile conditions)	Moderate–High (depends on substrate)	Requires sugars; energy and maintenance intensive	Arora et al. (2023); Bellini et al. (2022)
Food Waste/Glycerol	PHA, Starch blends	Low; uses existing waste streams	15–50% (*B. megaterium*, *Pseudomonas putida*)	Medium	Moderate	High in circular systems	Variable composition and regulatory barriers	Rivas-Castillo et al. (2024)

### Bio-composite polymers

**Fig. 1:**
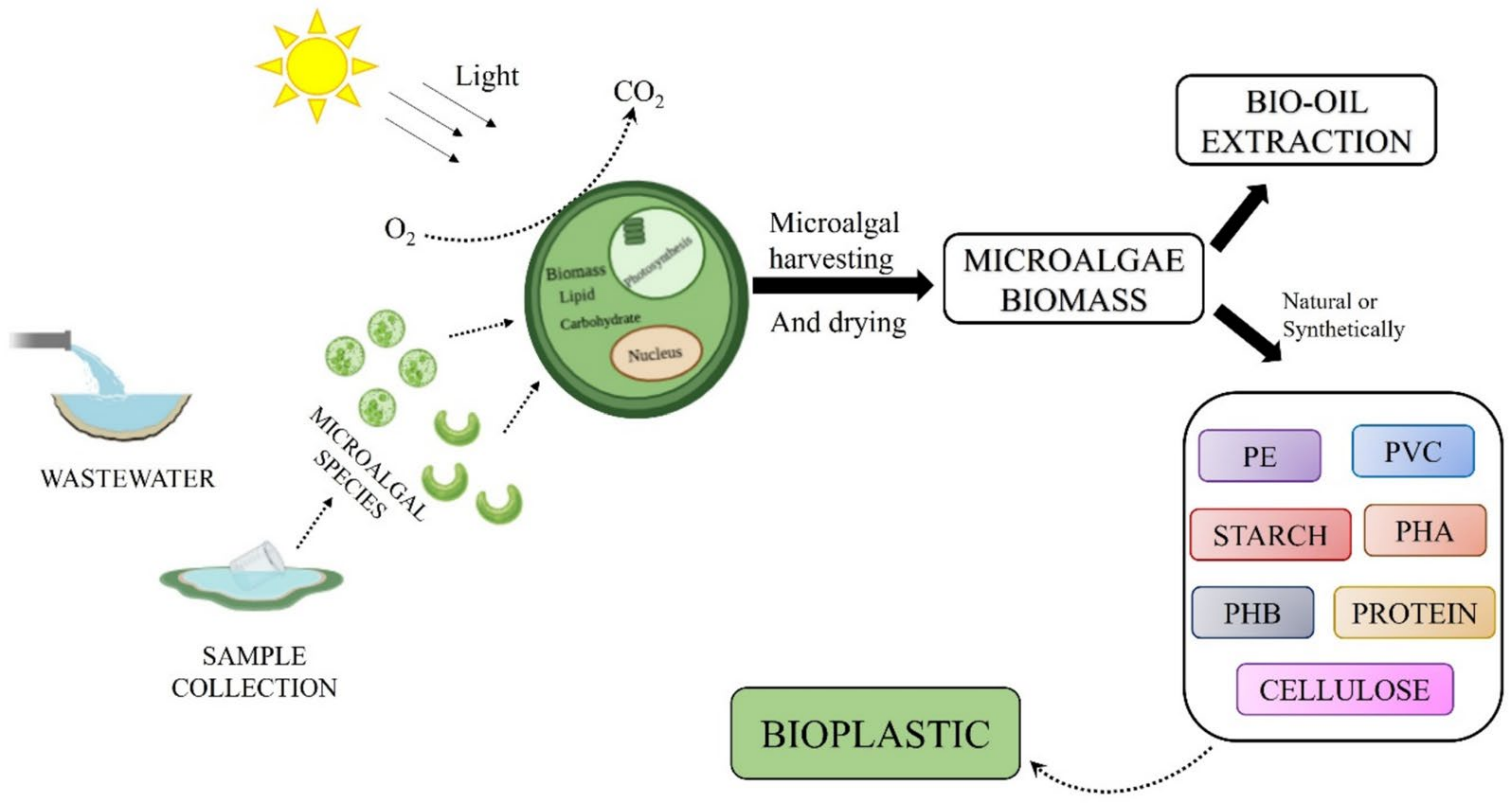
Microalgae as a sustainable producer of bioplastic

Bio-composites or bio-based polymers are gaining attention due to their biodegradability and mechanical strength. Various factors have an impact on the biodegradability of biopolymers, such as the use of raw materials, temperature, as well as a number of other conditions throughout the production process (Rai et al. 2021). These composites often combine algal biomass with biodegradable polymers to create materials suitable for food packaging and biomedical applications. Proteins, lipids, and secondary metabolites like polyphenols are a few examples of the biological sources from which the biopolymers are derived. Polybutylene adipate terephthalate (PBAT), PHB, and PLA are examples of conventional biodegradable compounds that are obtained from Cyanobacteria and microalgae by the process of fermentation (Karan et al. 2019). Microalgae can potentially lower the amount of carbon dioxide in the atmosphere and are carbon–neutral (Fig. 1). Microalgae serves as a dual role as fillers and reinforcing fibres in composites, effectively replacing a portion of petroleum plastic in blends, resulting in reduced costs and carbon footprints associated with polymer production (Bulota and Budtova 2015; Mihranyan et al. 2011). It has been found that *Chlorella* serves as an effective filler for PVC, enhancing composite volume, reducing density, and enhancing tensile strength (Zhang et al. 2000). A study examined corn starch bio-composites derived from *Scenedesmus*, *Nannochloropsis,* and *Spirulina*, revealing a 54% decrease in water vapour permeability upon adding microalgae. *Nannochloropsis* containing bio-composite exhibited enhanced oxygen permeability and matrix stiffness (Fabra et al. 2018). *Chlorella*-based algae bioplastic exhibited superior bioplastic behaviour, whereas *Spirulina*-based bioplastics showed improved blend performance (Zeller et al. 2013). The addition of algal biomass to PLA and PVA matrices significantly improves tensile strength and water resistance (Ng et al. 2024). Similarly, Dakhili et al. (2025) demonstrated that incorporating microalgal fillers enhances thermal stability and oxygen permeability of bio-composites, making them viable for packing application (Dakhili et al. 2025). Research continues to explore novel bio-composites using algal biomass as fillers or reinforcing agents.

### Poly lactide and polyalcohol

Polylactic acid is a thermoplastic polyester made through the fermentation of algal-derived lactic acid. It is FDA-approved for food packaging due to its biodegradability and low carbon footprint (Ghasemlou et al. 2024). It is one of the most effective plastics since it produces biodegradable plastic with a smaller amount of feedstock (sugar). The amount and rate of CO_2_ emission are also less than those of other biopolymers (Kaparapu 2018). It is one of the polymers that can easily change its stereochemical structure to acquire a larger molecular weight, and the variable isomers can change other physical properties like amorphousness and semi-crystallinity (Reddy et al. 2013). The PLA’s monomer is chiral and has two optical isomeric states as a result, numerous PLA structures can be constructed. The three main structures are Poly (L-Lactide) (PLLA), Poly (D-Lactide) (PDLA), and Poly (D, L-Lactide). It is possible to make PLA-biomaterials from a variety of algal compositions, which can then be applied to tissue applications. They are now widely used to create bio-composites. The polyalcohol substance known as polyvinyl alcohol, or PVA, is employed as an emulsifier, sizing agent, and protective film. Bio-composite PVAs offer the potential to develop novel material compositions (Onen Cinar et al. 2020). Recent advancements include the immobilization of *Lactobacillus plantarum* in PVA to boost lactic acid production, thereby reducing costs and improving yield (Chen et al. 2020). Moreover, incorporating microalgae as a renewable feedstock not only provides a sustainable carbon source but also reduces CO_2_ emissions during production. Microalgae contain high levels of fermentable carbohydrates, making them an efficient raw material for lactic acid synthesis. This strategy not only improves production efficiency but also supports environmentally friendly bioprocessing, with potential applications in bioplastics, pharmaceuticals, and food industries (Chen et al. 2020).

## Technologies for converting algal biomass into bioplastic

Algae are photosynthetic organisms that play an essential role in maintaining a healthy ecology by generating highly concentrated metabolites with versatile commercial applications (Rahman 2020). Among these applications, bioplastics, particularly PHAs and PLA, have gained attention. These metabolites are naturally produced by algal cells, but their efficiency can be enhanced through specific chemical treatments, genetic modifications, or alterations to growth conditions.

### Bio-composite additives- plasticizers and compatibilizers

Microalgae are microscopic and sensitive to moisture, which can limit their direct application as bio-composites. To enhance their processability, mechanical properties, and applicability, microalgal biopolymers are often combined with additives, plasticizers, and compatibilizers (Table 3). Recent advancements in bio-composite technologies focus on improving scalability, cost-effectiveness, and mechanical performance through optimized blending strategies (Onen Cinar et al. 2020). Plasticizers are crucial for enhancing the flexibility, biodegradability, and thermoplastic properties of biocomposites (Nanda and Bharadvaja 2022). They act by reducing the intermolecular forces between polymer chains, thereby increasing chain mobility. Commonly used plasticizers include glycerol, the most common one, sorbitol, 1,4-butanediol, and PEG (Onen Cinar et al. 2020). A recent study highlighted the use of PEG-300 as a plasticizer in *Kappaphycus alvarezii*-based biofilms, significantly improving flexibility and durability (Sudhakar et al. 2021). Additionally, glycerol remains the most effective plasticizer for algal-based bioplastics due to its availability, cost-effectiveness, and compatibility with various algal biopolymers (Gurunathan et al. 2025). However, it was observed that increasing the plasticizer concentration inversely affects the hardness of the bio-composite (Zeller et al. 2013).

**Table 3:** : Processing technologies and material properties of algal based bioplastics

Microalgae strain	Production technology	Optimum conditions	Methods for testing	References
*Spirulina platensis*	Hot compression mold	Plasticizer- glycerol 1,4-butanediol octanoic acid Polymer blend- biobased wheat Thickness- 1 mm Temperature- 120 °C Pressure- 40 bar Final product- Blended film	• Thermogravimetric analysis • Optical contact angle test • Thermal transition test • Scanning electron microscopy	Ciapponi et al. (2019)
*Chlorella vulgaris*	Hot compression mold	Plasticizer- glycerol Compatibilizer- MA 2–6% Polymer blend- PVA Size- 10 × 20 cm Temperature- 120 °C Pressure- 40 bar Final product- Blended film	• Scanning electron microscope • Mechanical properties	Khalis (2018)
*Chlorella* and *Spirulina*	Hot compression mold	Plasticizer- glycerol 1,4-butanediol octanoic acid Polymer blend- PE polymer Temperature- 150 °C Pressure- 24 ton Final product- Blended film	• Thermogravimetric analysis • Scanning electron microscope • Dynamic mechanical analysis • Mechanical properties	Zeller et al. (2013)
*Eucheuma Conttonii*	Handmade blended sheet	Latex- Artoarpus altilis and calostropis gigantea Dimensions- 210 mm X 297 mm Drying method—sun drying Final product- Blended film	• Tensile test method	Machmud et al. (2013)
*Chlorella* sp.	Solvent casting	Polymer blend—PVA Casting temperature—80 °C Dimensions—15 cm X15cm End product- Blended film	• Mechanical properties • Thermogravimetric analysis • Scanning electron microscopy • FTIR analysis	Sabathini et al. (2018)

Compatibilizers enhance the homogeneity and mechanical integrity of polymer blends by promoting interfacial adhesion between hydrophilic and hydrophobic components. Maleic anhydride, polyethylene-co-glycidyl methacryloyl carbamate, and diethyl succinate are commonly used compatibilizers that improve the compatibility between biopolymers and synthetic polymers (Zhu et al. 2017). The researcher altered the maleic anhydride content after finding that the PVA-Chlorella vulgaris biofilm's homogeneity and flexibility can be significantly improved by a compatibilizer (Gozan et al. 2018). They discovered that the tensile strength and elongation both increased with an increase in the compatibilizer quantity. The reason is that additional bonds are generated and strengthened between the hydrophobic anhydride group of the compatibilizer and the hydrophilic hydroxyl group in the microalgae as a result of their interaction (Gozan et al. 2018). In a study, *Artocarpus altilis’* red algal bioplastic was blended with the tropical plant’s latex, which significantly decreased the material's ductility. Further, red algal bioplastic with latex tensile strength was compared to *Calostopis gigantea's* tensile strength (Machmud et al. 2013). The result demonstrates that bioplastic from red algae combined with latex material still has a higher maximum tensile strength than those plastics made of starch (Machmud et al. 2013). Furthermore, algal bio-composites are benchmarked against other bio-composites such as Polybutylene adipate terepthalate (PBAT) and PLA blends to understand scalability and performance. Fayyazbakhsh et al. (2025) discuss that incorporating eco-friendly additives not only enhances performance but also reduces environmental impact. The inclusion of wool fibres and iconic liquid-based plasticizer shows promise in producing scalable and cost-effective bio-composites (Patrucco et al. 2024). A recent review emphasizes that the combination of waterborne acrylic dispersion with algal biopolymer significantly improved the mechanical properties of the resulting bio-composites (Solera-Sendra et al. 2025). These findings align with the growing interest in integrating sustainable additives that offer enhanced durability and reduced production costs. Despite these advancements, challenges persist in achieving commercial scalability. High production cost, variability in mechanical properties, and moisture sensitivity limit the practical application of algal bio-composites. Addressing these issues requires the development of more efficient plasticizers and compatibilizers tailored specifically for microalgal polymers. To overcome these challenges, recent research highlights several upscaling approaches. Di Caprio et al. 2023 optimized cultivation parameters to enhance starch yield in *Tetrasekmis suecica*, improving its downstream conversion to PLA.

### Genetic engineering approach to plastic production from algae and cyanobacteria

**Fig. 2:**
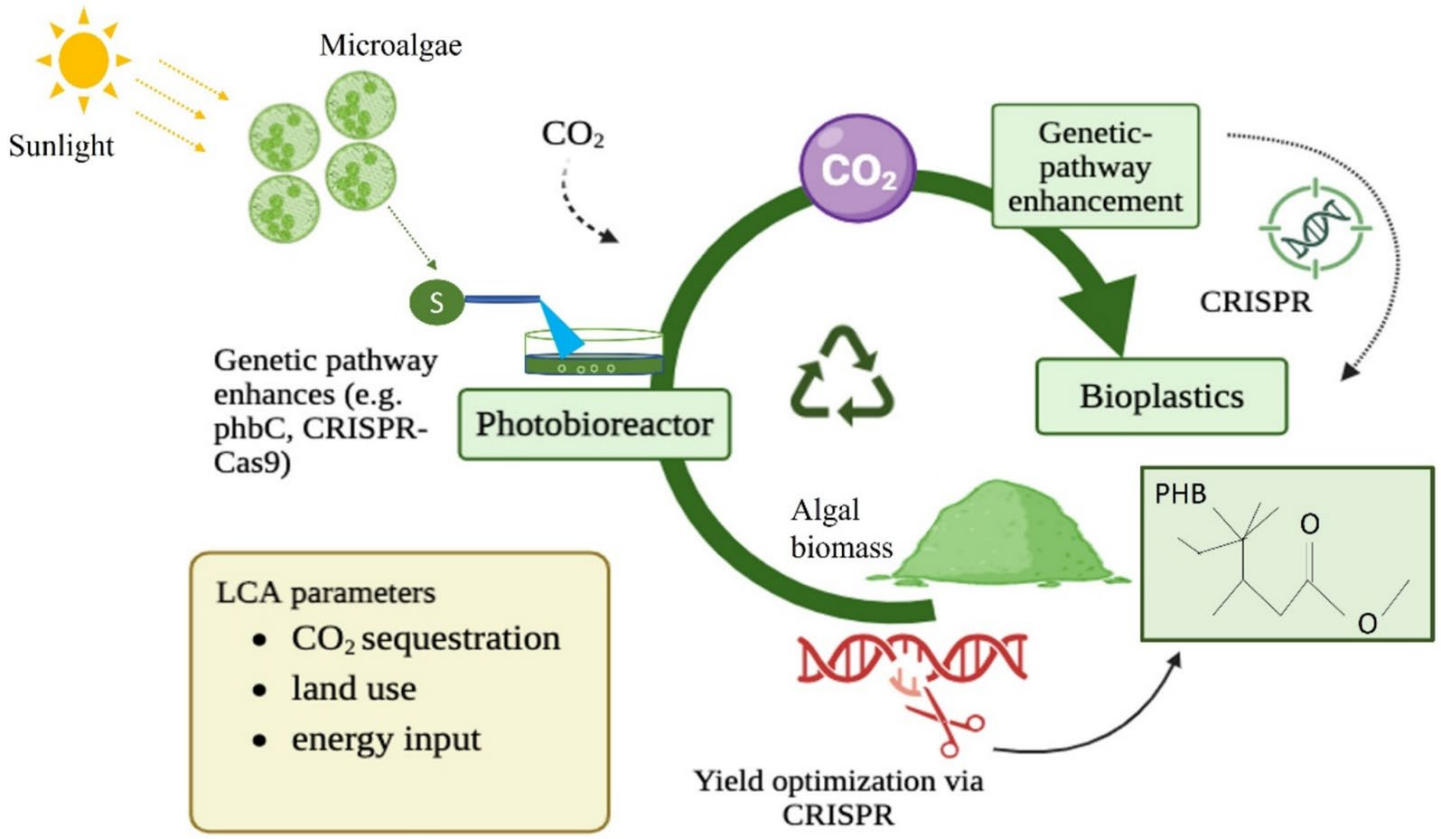
Schematic bioplastic production from Genetically Modified microalgae

Recent advances in genetic engineering have significantly enhanced the potential of algae and cyanobacteria for bioplastic production, especially in synthesizing PHAs (Nawaz et al. 2024). These developments include the insertion of optimized metabolic pathways, regulatory elements, and heterologous genes that enhance both yield and polymer quality (Fig. 2) (Carbonell 2021). Third-generation bioplastics, often referred to as algal bioplastics, focus on utilizing these organisms due to their simplicity and accessibility for genetic manipulation. Notably, *Chlamydomonas reinhardtii*, *Synechococcus* sp., and *Synechocystis* sp. are prominent cyanobacterial strains that have been successfully engineered to produce PHAs (Koller 2020). Genetic engineering techniques allow for the insertion of bacterial genes responsible for PHA synthesis into algal genomes. For instance, Chaogang et al. (2010) modified *C. reinhardtii* with expression vectors containing the phbB and phbC genes from *Ralstonia eutropha*, resulting in the accumulation of PHB granules within transgenic algal cells. Similarly, Hempel et al. (2011) introduced the bacterial PHB biosynthesis pathway into the diatom *Phaeodactylum tricornutum*, achieving PHB levels of up to 10.6% of algal dry weight while maintaining a low-cost and environmentally friendly approach. Cyanobacteria, due to their simple genetic makeup, are highly amenable to metabolic engineering for enhanced polyhydroxybutyrate (PHB) production (Koksharova and Wolk, 2002). Among cyanobacterial strains, *Synechocystis* sp. PCC 6803 has been the most extensively studied, benefiting from decades of research on photosynthesis and genetic annotations (Koksharova and Wolk 2002; Wilde and Dienst 2011). PHB biosynthesis in cyanobacteria involves three key enzymatic steps, where acetyl-CoA is converted into PHB through the action of *phaA*, *phaB*, and *phaC* genes (Hauf et al. 2015). While genetic modifications, such as introducing *PHA synthase* from *C. necator*, have improved enzymatic activity, overall PHB accumulation remains a challenge (Sudesh et al. 2002). Recent strategies, including overexpression of *phaAB* and acetoacetyl-CoA reductase, have led to increased R-3-hydroxybutyrate production, with the highest volumetric productivity reported at 263 mg L⁻1 d⁻1 (Carpine et al. 2017). Alternatively, random mutagenesis has been employed to generate improved cyanobacterial strains, with UV-induced mutations in *Synechocystis* sp. PCC 6714 enhancing PHB content up to 37% (DCW) by altering phosphate transport proteins (Kamravamanesh et al. 2018) (Table 4). Despite promising yields in lab settings, scaling up genetically modified strains remains challenging. A major hurdle in maintaining gene stability and consistent bioplastic yields under industrial-scale conditions. Variation in light, temperature, and nutrient availability can lead to reduced gene expression and elevated metabolic stress (Agarwal et al. 2022). For instance, *Synechococcus* sp. PCC 7942 was engineered to improve CO_2_ utilization efficiency, directly enhancing the PHA synthesis pathway (Agarwal et al. 2022). Furthermore, it has been shown that heterologous expression of the bacterial PHA synthase gene in microalgae enhances PHA accumulation. However, maintaining cultivation conditions remains a challenge (Diankristanti et al. 2024). CRISPR-Cas9 and CRISPR-Cas12a tools have enabled targeted knock-ins and knockouts of biosynthetic and regulatory genes (Beher et al. 2018). This genetic precision enhance carbon flux towards PHB and PHA biosynthesis pathways. For example, CRISPR-based deletion of polyphosphate kinase and glycogen synthase gene in *Synechocystis* sp. PCC6803 increased intracellular acetyl-CoA pools, enhancing PHA accumulation while maintaining cell viability (Yao et al. 2020). In *Chlamydomonas reinhardtii,* multiplexed CRISPR edits of citrate synthase and malate dehydrogenase genes redirected TCA fluxes towards PHA synthesis, leading to a ≥ 40% increase in PHB content (Nawaz et al. 2024). Emerging strategies now employ multi-gene CRISPR arrays and synthetic riboregulators to simultaneously modulate entire metabolic pathways. In a recent breakthrough, *Synechococcus elongatus* engineered with CRISPR activation of RuBisCO and heterologous expression of bacterial phaCAB operon achieved a productivity rate of 263 mg L⁻^1^ d⁻^1^ in photobioreactor setups (Carpine et al. 2017). However, achieving consistent yields under industrial-scale conditions remains a challenge. The integration of a multi-omics approach has further improved genetic constructs, enabling more efficient bioplastic production (Kuo et al. 2022). Moreover, the economic feasibility of using genetically engineered algae remains contentious. Wild CRISPR-based modifications can significantly boost PHA production, but the cost of maintaining genetically stable strains, ensuring biosafety and scaling up the production process can be prohibitive. A techno-economic analysis of CRISPR-modified cyanobacteria indicated that the operational costs of maintaining sterile conditions and preventing genetic drift could undermine commercial viability (Mogany et al. 2024). While genetically modified algae typically yield higher PHA and PHB content, they are often costly and face more stringent regulatory hurdles compared to naturally occurring high-yield strains like *Chlorella pyrenoidosa*. GM strains such as *Synechococcus sp.* are engineered to enhance carbon fixation and bioplastic synthesis, while non-GM strains can be more adaptive to varying cultivation conditions (Agarwal et al. 2022). The choice between GM and non-GM approach largely depends on the specific application context and the desired yield-to-cost ratio. To address these challenges, researchers are exploring the use of synthetic biology frameworks that integrate gene circuits capable of dynamic regulation, allowing for better adaptation to changing environmental conditions. Additionally, the development of bioprocessing strategies that include integrated cultivation and extraction systems may help reduce operational costs, as suggested in recent studies (Sharma et al. 2024a; b). Despite these advancements, concerns regarding the ecological impact of genetically modified organisms (GMOs) persist. Genetic engineering offers a promising approach to enhancing the production of algal bioplastics. Advancing research on metabolic pathway optimization, strain stability, and environmental impact mitigation will be essential to unlocking the full potential of algae as a sustainable bioplastic feedstock.

**Table 4:** Culture and Genetic variables affecting PHB yield in Recombinant Cyanobacteria and Microalgae

Recombinant microalgae	Culture conditions	Genetic modifications	PHB content	Key bottlenecks or yield determination	References
*C. reinhardtii*	Photoautotrophic	Introduction of phbB and phbC genes from *R. eutropha*	6 µg/g	limited expression of only two PHB pathway genes; potential lack of precursor (acetyl CoA)availability under photo auto trophy	Chaogang et al. (2010)
*Synechocystis sp.* PCC6803	Photoautotrophic	Double PTA (phosphotransacetylase) and ACH (acetyl-CoA hydrolase) deletion and heterologous overexpression of phosphoketolase (xfpk) from *Bifidobacterium brevephosphoketolase*	12.4%	Enhanced carbon flux through heterologous phosphoketolase pathway; suggests flux redistribution improved acetyl-CoA availability	Carpine et al. (2017)
*Synechocystis sp.* PCC 6803 (GOX50)	depletion of N2 sources naturally	SigE overexpression	1.4 mg/100 mg	Nitrogen limitation affects global metabolism; SigE regulates sugar catabolism, but may not sufficiently direct flux to PHB	Osanai et al. (2013)
*Synechocystis sp.* PCC 6714 (mutant MT_a24)	starvation of phosphorus and nitrogen	Point mutation in integral membrane protein A of the phosphate-specific transport system caused by random UV mutagenesis (PstA)	37%	Phosphate uptake disruption may activate PHB accumulation as a stress response	Kamravamanesh et al. (2018)
*Synechocystis sp.* PCC6803	Photoautotrophic; 21-day batch; phosphate limitation	Slr1829 and Slr1830 deletion	533.4 mg/L	Disruption of carbon storage regulatory genes enhances carbon flux towards PHB under phosphate limitation	Wang et al. (2013)
*Phaeodactylum tricornutum*	Autotrophic; NO3-induced synthesis of PHA	R. eutropha H16 PHA production gene insertion	10.6%	Nitrogen induction supports PHA biosynthesis, but native carbon flux in diatoms may limit polymer yield	Hempel et al. (2011)
*Synechococcus elongatus* UTEX 2973	Photoautotrophic	insertion of the phaABC operon from C. necator	16.7%	Fast growing chassis; PhaABC expression enables high yield; efficient precusor generation; supports improved PHB	Roh et al. (2021)
*Synechococus elongatus* PCC 7942	Photoautotrophic	insertion of the phaA and phaB genes from C. necator, tesB from E. coli and P. putida, and nphT7 from Streptomyces species as well as thioesterase genes	1.2 g/L	Multi gene engineering addresses multiple bottlenecks; tailored expression of thioesterases enhances precursor availability	Ku and Lan (2018)

## Applications of bioplastics

**Fig. 3:**
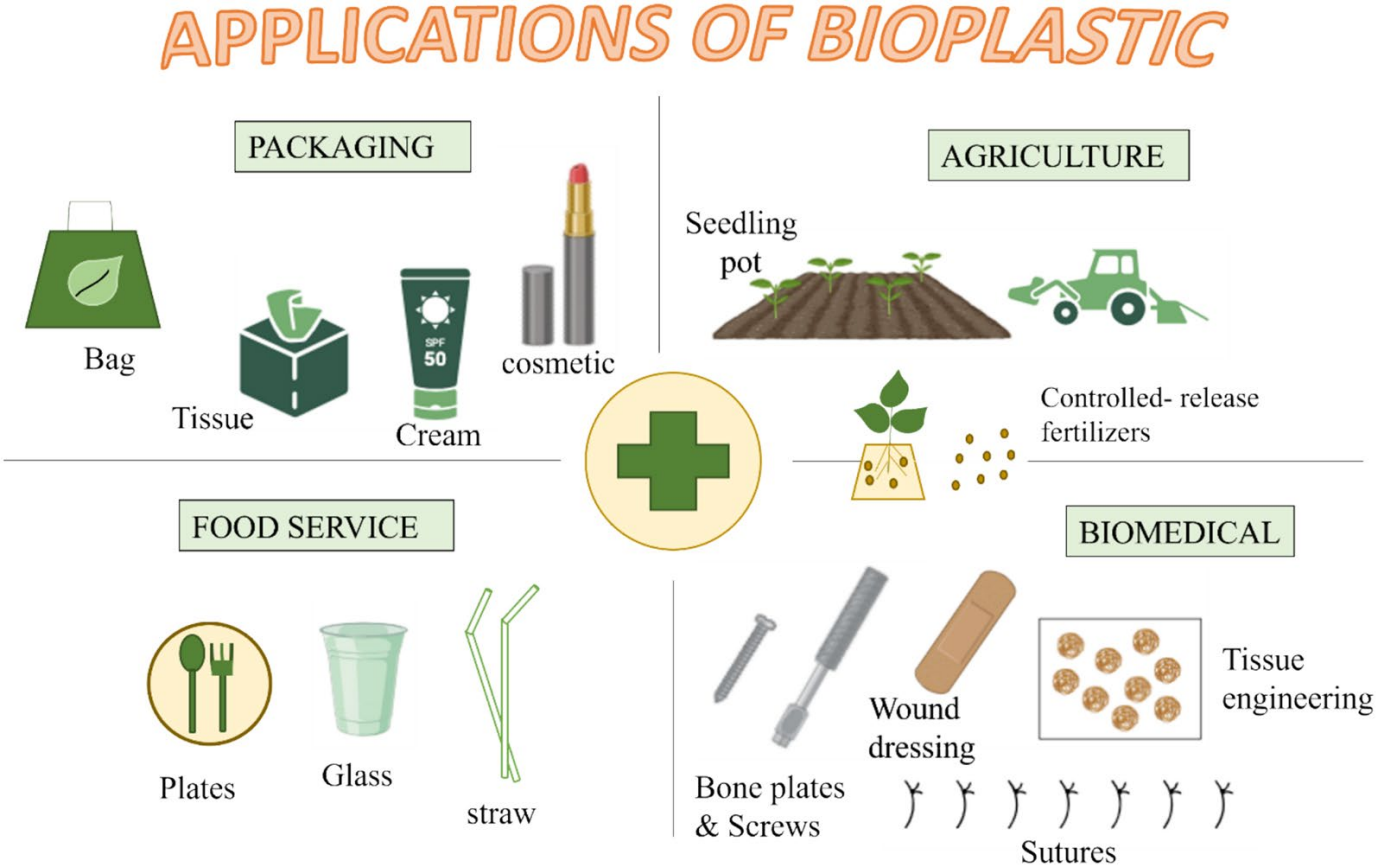
Various applications of bioplastic

Bioplastics are polymers derived from renewable biological resources and are characterized by their biodegradability. These materials are increasingly recognized as vital replacements for conventional petrochemical plastics, the disposal of which poses significant environmental challenges. The successful development and commercialization of bioplastics present a unique opportunity to advance sustainability and contribute positively to the environment. Various applications of bioplastics are illustrated in Fig. 3.

### Food packaging

The increase in global population, consumerism, and food demand has intensified the necessity for effective packaging solutions. Packaging is essential for maintaining the physical, chemical, and microbiological integrity of food products. Traditional plastics have long been used to ensure the safe packaging and delivery of food, and growing awareness of sustainability has led to an increased interest in recycled and renewable materials (Peelman et al. 2013). Bioplastic packaging has emerged as a promising alternative, offering properties such as biodegradability, lower carbon footprint, and potential compostability. Products made from bioplastic include toys, cups, plates, lamination films, cutlery lids, and containers. Key performance metrics for evaluating biopolymer-based food packaging include moisture permeability, oxygen permeability, mechanical strength, and durability. Several studies have explored the potential of various microalgae species, including *Scenedesmus*, *Spirulina platensis*, *Nannochlorosis gaditana*, *Palmaria palmata*, *Chlorella* sp., *Himanthalia elongata*, and *Porphyra columbina* for producing edible films and coatings (Albertos et al. 2019; Martínez-Sanz et al. 2020). These microalgae are rich in polysaccharides and proteins, which enhance their thickening properties, making them suitable for biodegradable food packaging and coating materials. For instance, Scenedesmus-derived films exhibit excellent oxygen barrier properties, critical for packaging dairy products and preventing oxidation and microbial growth (Coppola et al. 2021).

PLA synthesized from renewable sources like corn starch or sugarcane, is widely used as a green packaging polymer due to its mechanical strength, durability and thermal flexibility. It has high resistance to high temperatures compared to conventional synthetic polymers such as polyethylene terephthalate and polystyrene. PLA offers minimal production costs, eco-friendly biodegradation, high durability, thermal plasticity, and aroma resistance, making it a viable alternative for food packaging (Nanda et al. 2022). Another significant bioplastic, Poly-3-hydroxybutyrate (PHB), is produced via microbial fermentation and is valued for its biocompatibility and biodegradability (Mlalila et al. 2018). It is used in applications such as food supplement encapsulation and packaging, and in the manufacture of diapers and bottles (Mlalila et al. 2018).

Starch-based biodegradable materials offer a sustainable alternative to conventional plastics due to their biodegradability and renewability. Despite challenges related to water sensitivity and mechanical performance, advancements in polymer blending and plasticizers incorporation have significantly improved their usability (Jiang et al. 2020; Onyeaka et al. 2022). Innovative combinations with polyvinyl alcohol (PVA) and chitosan have further enhanced their moisture resistance and tensile strength (García-Guzmán et al. 2022).

Protein and lipid-based biopolymers can be produced from animal or plant proteins, such as soy protein, whey protein, collagen, or gelatin offer better mechanical strength and aroma resistance compared to polysaccharide-based polymers. (Garrison et al. 2016). Additionally, lipid-based biopolymers consist of glycerides, terpenes, and fatty alcohols, are applied as a moisture barrier in packaging, especially for meat, fruits, and vegetables. These films can also incorporate essential oils for antimicrobial activity (Rodrigues et al. 2016).

### In agriculture

In agriculture, bioplastics are employed as nets, mulch films, and grow bags. These materials help to improve crop yield and reduce weed growth, and prevent pest infestations. Conventional synthetic polybags are being replaced by bioplastic grow bags derived from PHAs due to increasing sustainability awareness. These bags are non-toxic, biodegradable, and do not damage roots (Coppola et al. 2021), while maintaining soil structure by retaining moisture and preventing contamination. An emerging innovation is the use of Solaplast films, made from algal bioplastics, to shield crops like banana and grapevine bushes from dust. After usage, these films’ innovative application involves bio-based plant pots and baskets, which support root growth and moisture retention, providing a sustainable solution in horticulture (Ashter 2016).

### In the biomedical field

Bioplastics in the biomedical field address the need for biocompatibility, non-toxicity, and biodegradability. Hospitals and clinics require thoroughly sterilized needles and non-reactive equipment that produces minimal interference with diagnostic or treatment procedures. Conventional synthetic plastic is cheap, convenient, reliable, and non-reactive, but contributes to significant hospital waste. Biodegradable dental implants are now widely used to fill cavities after tooth extraction, and non-toxic, easily biodegradable sutures are developed by surgeons for operations, maintaining strength and durability (Ashter 2016). These sutures dissolve and metabolize within the body, leaving no trace. Biodegradable materials, such as biodegradable tyrosine-derived polyarylate, are used to create tissue regeneration membranes and absorbable bactericidal envelopes. PolyActive and OctoDEX are biodegradable polymers used in drug delivery technologies for site-specific drug delivery (Ashter 2016), providing controlled release of proteins and lipophilic small molecules for local and systemic administration due to their biodegradability and linear release features (Ashter 2016). Furthermore, PHAs are utilized across domestic, agricultural, and medical fields, including in nanoparticles for drug delivery, biocompatible porous implants, antibacterial applications, and tissue engineering scaffolds (Albuquerque et al. 2018). Recent advancements in algae-based biomaterials have also shown promise in biomedical applications, offering sustainable and functional alternatives for drug delivery systems, wound healing, and regenerative medicine (Iqbal et al. 2022).

### In household articles

Conventional plastics derived synthetically from non-renewable resources are being replaced by bioplastics, which are eco-friendly alternatives that degrade naturally and quickly. They produce fewer greenhouse gases and contribute to a more sustainable environment. Bioplastics offer almost the same properties as synthetic plastics but may have slightly lower mechanical strength (Coppola et al. 2021). However, strengthening them is possible through methods such as dehydrothermal treatments and ultrasound applications (Jiménez-Rosado et al. 2020), further expanding their potential applications. Biodegradable shopping bags made from starches combined with biodegradable polymers such as PLA or BASF EcoFlex have been developed by Grab-n-Go, meeting ASTM compostable standards. Disposable biodegradable plastics are replacing polystyrene and polyolefin in houseware articles such as cups, storage containers, and toys, with brands like Nature-Ware and United Colors of Benetton manufacturing compostable household items (Ashter 2016).

### As antioxidants

In the food industry, Biopolymers are utilized as antioxidants due to their safety compared to synthetic antioxidants (Sivakanthan et al. 2020). Gelatine sodium caseinate molecules have substantial antioxidant activity, and bioactive peptides derived from cow or goat milk exhibit high antioxidant properties, enhancing shelf life (Zanutto-Elgui et al. 2019). Additionally, bioactive peptide, *Amphiroa rigida*, an alga strain, has shown potential in pharmaceutical applications due to its antioxidant capacity (Gopu and Selvam 2020).

## Challenges of algal bioplastics

While algae hold substantial promise as a renewable feedstock for bioplastic production, several challenges impede their widespread commercialization and application. These obstacles range from strain selection and polymer optimization to environmental concerns and economic viability, wastewater management, and regulatory limitations. Addressing these challenges is crucial to developing cost-effective, scalable, and sustainable algal bioplastic production.

### Optimization parameters in algal bioplastic production

With growing interest in sustainable materials, optimization parameters in algal bioplastic production—such as strain selection, nutrient modulation, and polymer yield—are increasingly informing broader bioresource utilization strategies (Sharma et al. 2024a; b). These insights are particularly relevant to algal biofuel systems, where similar constraints in biomass productivity, processing efficiency, and economic viability apply. As the global energy sector intensifies its shift toward low-carbon alternatives, microalgae have emerged as a promising feedstock for sustainable biofuel production due to their high lipid content, rapid growth, and non-competition with food crops (Mishra et al. 2018). However, despite their potential, the commercialization of algal biofuels remains constrained by several technical and economic challenges. Downstream operations such as dewatering and lipid extraction can account for over 50% of total production costs, though recent pilot-scale innovations in wet extraction and integrated harvesting systems have demonstrated cost reductions of up to 20% (Barik et al. 2024). Pretreatment remains a bottleneck due to the energy demands of disrupting resilient algal cell walls, but 2024 studies show that combining mild hydrothermal methods with enzymatic hydrolysis can enhance lipid recovery by 30–40% while maintaining a favorable energy return. Yield optimization is further complicated by environmental variability and contamination risks in open systems; however, integrating direct air capture (DAC) with photobioreactors has shown promise, with techno-economic models projecting a minimum fuel selling price as low as $4.70 per gallon gasoline equivalent under favorable policy and carbon intensity scenarios (Bendle et al. 2024).

Selection of the most suitable microalgae strain for producing biopolymers remains a significant challenge due to its diverse metabolic capabilities and polymer synthesis potential among algal species. Different microalgae species exhibit varying capacities for synthesizing biopolymers with desired characteristics, such as *Himanthalia elongata*, *Porphyra columbina, Scenedesmus*, *Spirulina platensis,* etc. (Albertos et al. 2019; Martínez-Sanz et al. 2020). However, achieving optimal polymer composition to enhance strength and flexibility remains an ongoing research focus. The complexity arises from factors such as biodegradability, degradation rate, feedstock renewability, molecular weight, brittleness, and polymer size, which significantly influence the final material properties (Coppola et al. 2021). For example, *Scenedesmus obliquus* has shown potential for producing starch-rich biopolymers, but issues such as brittleness and low tensile strength hinder its practical application (Rai et al. 2021).

In large-scale operations, achieving optimal polymer content often requires precise control of nutrient limitation (e.g., nitrogen or phosphorus), which is difficult to maintain consistently in open ponds or outdoor systems due to fluctuating environmental factors such as temperature, light intensity, and contamination risks. These inconsistencies reduce yield predictability and compromise process efficiency (Penloglou et al. 2024). Optimizing cultivation parameters is critical for enhancing the yield and efficiency of PHA and PLA production from microalgae (Table 5). Key parameters influencing algal metabolism and biopolymer synthesis include light intensity, temperature, pH, photoperiod, nutrient limitation such as nitrogen and phosphorus, and carbon/nitrogen (C/N) ratios.

**Table 5:** Quantitative comparison of cultivation parameters and PHB yield among microalgal strains

Microalgae species	Light intensity (µmol·m⁻2·s⁻1)	C/N ratio	Stress condition	Temperature	Light:Dark	pH	Polymer type	PHB yield (% DCW)	References
*Chlorella vulgaris*	500	20:1	Nitrogen starvation	28	16:8	7–8	PHB	35	Selvaraj et al. (2021)
*Synechocystis* sp. PCC 6803	100	10:1	Phosphorous limitation	25–30	12:12	7	PHB	10.6	Koksharova and Wolk (2002); Wilde and Dienst (2011)
*Phaeodactylum tricornutum*	200	30:1	Sulphur deprivation	22	14:10	8	PHB	10.2	Hempel et al. (2011)
*Spirulina platensis*	300	15:1	Salinity stress	25–30	12:12	9	PHB	22	Albertos et al. (2019); Martínez-Sanz et al. (2020)
*Scenedesmus obliquus*	450	25:1	Nitrogen limitation	26	16:8	7.2	PHB	28	Kartik et al. (2021)
*Nannochloropsis gaditana*	250	18:1	High light exposure	24	18:6	7.8	PHB	16	Martínez-Sanz et al. (2020)

*Light intensity* and photoperiod play a critical role in optimizing carbon flux towards starch biosynthesis in microalgae. Di Caprio et al. (2021) demonstrated that optimizing cultivation conditions, including a 16:8 light/dark cycle and light intensity of 150 µmolm^−2^ s^−1^, significantly enhances the starch accumulation in *Tetraselmis suecica*. This optimized starch was subsequently recovered and converted into lactic acid, supporting downstream PLA synthesis (Di Caprio et al. 2021).

*Nutrient stress* especially nitrogen limitation, has been consistently linked with enhanced PHA accumulation in microalgae and cyanobacteria. In *Spirulina* sp. and *Synechocystis* sp*.*, nitrogen and phosphorus deprivation shift metabolic flux away from protein synthesis towards lipid and PHA accumulation (Venkatesh et al. 2024). Studies have demonstrated that under nitrogen-strained conditions, PHA content in cyanobacteria could increase by up to 40% of dry cell weight (Sudesh and Iwata 2018; Reddy et al. 2003).

*Temperature and pH* must be carefully controlled; for most microalgal strains, optimal PHB synthesis occurs at pH 7–8 and 25–30 °C. Selvaraj et al. (2021) demonstrated that maintaining neutral pH and modulating the C/N ratio could enhance both growth and biopolymer accumulation.

Emerging approaches such as photosynthetic metabolic flow redistribution, adaptive stress cycling and energy-efficient waste valorisation systems are now being explored to address industrial-scale production constraints (Wicker et al. 2021). These optimization strategies can help overcome metabolic bottlenecks and improve both yield and process economics.

### Addressing environmental impacts

Despite being marketed as eco-friendly, algal bioplastics may pose environmental risks during production, usage, and degradation. One major concern is the emission of greenhouse gases such as CO2 and methane during degradation, especially in anaerobic environments like landfills (Rasul et al. 2017). Studies have reported that PLA and PHB, although degradable, can produce methane emissions under certain conditions, contradicting their sustainability claims. In many municipal waste systems, especially in developing regions such as South and Southeast Asia, algal bioplastics are not separated from conventional plastic, leading to contamination and improper disposal. Moreover, the lack of industrial composting facilities capable of reaching the required degradation temperature further limits their environmental advantages (Lara-Topete et al. 2024). Moreover, the degradation rate of bioplastic can vary significantly depending on the environmental conditions. For instance, PLA requires a high temperature of 60 °C for effective composting, making it unsuitable for natural environments where low temperatures prevail (Kalita et al. 2021). There is also a risk of toxic by-products emerging from the degradation of some algae-derived polymers, especially if additives or chemical stabilizers are present. This necessitates the development of eco-friendly degradation pathways and comprehensive life cycle assessment (LCA) to ensure that the environmental benefits of algal bioplastics outweigh their drawbacks. Addressing these environmental challenges requires adopting integrated waste management strategies that promote safe degradation and recovery.

### Technical and economic production

Although algal bioplastics have shown promise at the laboratory scale, their transition to industrial-scale viability remains hindered by critical technological and economic barriers. One of the foremost issues is the cost of downstream processing, particularly in harvesting and drying microalgal biomass, which can contribute up to 40% of the total production cost in open pond or photobioreactor systems (Quiroz-Arita et al. 2022). In real operational settings, drying and dewatering steps are highly energy-intensive and cannot be economically sustained without integration with low-grade waste heat or process intensification strategies (Dutta et al. 2023). Moreover, chemical extraction of PHAs often generates hazardous by-products and imposes environmental trade-offs at scale (Goswami et al. 2022). Additionally, contamination from native microbial populations in open systems can drastically reduce polymer purity and increase separation costs. Pre-treatment technologies, especially for rigid algal cell walls, also impose challenges. Mechanism disruption (bead milling, ultrasonication) and chemical digestion (alkaline, enzymatic hydrolysis) used to extract intracellular PHAs and starch-based biopolymers often exhibit poor energy yield and environmental trade-offs at larger scales (Goswami et al. 2022). Optimization of biomass composition through nutrient limitation (e.g., N or P starvation) can enhance polymer accumulation, but it concurrently slows growth and overall productivity, creating a trade-off between biomass yield and polymer content (Watkins et al. 2024). Recent pilot-scale efforts have yielded mixed outcomes. For instance, a techno-economic analysis (TEA) was conducted by Witkowski et al. (2022) for algae. It was cultivated in anaerobic effluent, estimated PHA production cost between $7 and to 9 per kg, which is still uncompetitive compared to petroleum-derived plastic. However, integrating biorefinery models, Co-producing biofuels, pigments, and proteins can improve profitability and reduce waste (Narayanasamy et al. 2024). Additionally, biofilm-based cultivation systems have demonstrated the ability to significantly reduce water and energy input while enabling continuous harvesting (Watkins et al. 2024). Beyond engineering, policy and market support mechanisms such as renewable carbon credit, carbon pricing, and public procurement mechanisms could significantly influence commercial uptake. Life cycle assessment and resource harmonization framework suggest that with better strain selection, CO_2_ capture integration and valorisation of non-polymer co-products, algae-based power plastics may soon approach techno-economic parity with starch and sugar-based bacterial systems.

### Effective waste management

Managing bioplastic waste effectively is critical to ensure its environmental sustainability. Unlike conventional plastics, bioplastics often require specific conditions for degradation, such as industrial composting facilities. While composting remains the most viable disposal method, not all bioplastics are certified compostable, and the degradation rates can vary widely. For example, PHA-based films degrade faster under aerobic composting than under anaerobic landfill conditions (Chidambarampadmavathy et al. 2017). Improving waste management requires establishing standardized composting facilities and promoting biodegradable waste collection systems. Moreover, the absence of standardized biodegradability certification frameworks in several regions limits accurate labelling of algal bioplastic. This leads to improper waste categorization and exclusion from industrial composting systems, reducing disposal efficiency (Jankov 2018).

### Regulatory and market limitations

Regulatory frameworks are essential for the commercialization of algae bioplastics. However, the lack of standardised certification for biodegradability, compostability, and bio-based content can confuse consumers and manufacturers. Countries vary significantly in their regulatory approaches, leading to inconsistent standards and market barriers. Moreover, market acceptance is hampered by consumers'scepticism about bioplastic performance and higher cost compared to conventional plastics. Encouraging public–private partnerships, government incentives, and clear labelling practices can boost consumer confidence and expand market uptake (Marques and Berg 2020). Establishing comprehensive policies that support research, development, and commercialization while addressing socio-economic impact will be crucial for integrating algal bioplastic into the circular economy.

## Role of algal bioplastic in the green economy

Microalgae are increasingly being recognised as a promising alternative for the production of non-renewable products due to its versatility and sustainability (Alam and Wang 2019). Unlike bacterial counterparts, microalgae are particularly advantageous due to their adaptability in cultivation, use of waste as a feedstock, and natural environmental conditions. This adaptability makes them viable for large-scale applications, aligning well with the principles of the green economy. However, the transition from laboratory to industrial scale remains challenging. The most significant obstacle lies in the capital expenses associated with downstream processing, including harvesting, dewatering, extraction, and bioplastic synthesis. This process requires significant energy input and advanced technological setups, which drive up the production cost. Furthermore, the management of wastewater and disposal remains a persistent challenge, as improper handling can lead to environmental contamination and reduce the overall sustainability quotient. The concept of microalgal biorefinery is evolving as a sustainable model to integrate the production of fuels, polymers, feedstocks, and pharmaceuticals. In this context, microalgae can act as a cost-effective building block for biorefineries, much like crude oil, which, through fractionation, produces a range of products at each stage of distillation. This analogy highlights the potential economic viability of integrating bioplastic production within a broader bio refinery framework. One strategic approach is to utilise algal biomass after extracting high-value compounds like pigments, fatty acids, and lipids. The remaining biomass, rich in carbohydrates and proteins, can then be employed for bioplastic production. This not only optimises resource utilization but also lowers the overall cost of algal cultivation. For instance, in biodiesel production, after lipid extraction, the residual biomass can be combined with by-products glycerol, to serve as a plasticizer in the synthesis of bioplastics. This synergistic approach not only minimises waste but also supports a closed-loop production system, where the by-products of one process act as feedstock for another.

Algal bioplastics contribute significantly to the green economy by utilising CO_2_ emissions as a carbon source during photosynthesis, thereby mitigating atmospheric CO_2_ levels. Unlike conventional plastics, which contribute to greenhouse gas emissions throughout their life cycle, algae bioplastic plays a dual role, i.e., in reducing CO_2_ emissions through bio fixation during growth and minimizing environmental pollution during degradation, as they are inherently biodegradable. Moreover, the integration of algal bioplastics into urban CO_2_ management systems, such as photobioreactors in industrial areas, can further enhance their role in carbon capture. By closing the carbon loop, algal bioplastics help build a circular and sustainable bioeconomy. To realize the full potential of algal bioplastics in the green economy, it is essential to reduce production costs through innovations in cultivation techniques, downstream processing and biomass valorisation. Establishing policies that incentivize bioplastics production, coupled with consumer awareness campaigns, can accelerate market adoption. Moreover, employing techno-economic analysis (TEA) and life cycle assessment (LCA) helps in evaluating the environmental benefits over conventional plastics (Rahman et al. 2020).

By adopting integrated biorefinery models, where multiple products are derived from a single biomass source, the economic viability of algal bioplastics can be significantly enhanced. This approach not only supports the circular economy but also contributes to the green economy by reducing resource wastage and promoting eco-friendly manufacturing practices.

## Future perspective

Microalgae hold immense potential in addressing global plastic pollution due to their ability to biodegrade plastic waste through enzymatic activity. These enzymes facilitate the breakdown of plastic polymer, converting it into metabolites like carbon dioxide, water, and new biomass. Exploring how microalgae process plastics into useful compounds remains a promising area of research, as understanding these metabolic pathways could revolutionize plastic waste management (Shamsuddin et al. 2017). Despite this potential, the transition from laboratory findings to industrial applications remains challenging. Addressing societal and governmental concerns is crucial, as integrating sustainable bioplastic solutions into commercial sectors such as healthcare, food, nutraceutical, and cosmetic requires holistic approaches that consider both environmental and economic factors.

In parallel, the environmental risks of genetically modified (GM) algal strains demand scrutiny. While metabolic and genetic engineering can significantly improve yields of PHAs and PLA, the introduction of GM microalgae into open environments could pose unforeseen risks to ecosystems, including gene flow, toxin production, and microbiome disruption. Therefore, future research must prioritize ecological risk assessments and develop bio-contaminant strategies before deploying engineered strains at scale (Yadav and Nikalje 2024). A critical gap in the current research is the lack of standardized lifecycle assessments (LCAs) for algal bioplastics. LCAs are essential tools for evaluating the sustainability and ecological footprint of bioplastic production from microalgae, covering the entire supply chain from biomass cultivation to degradation. Although preliminary LCAs exist, they vary widely in methodology and assumptions, limiting their comparative and policy-making utility (Fiorentino et al. 2023). Harmonizing LCA protocols and integrating them into regulatory frameworks can ensure transparent benchmarking of algal plastics against petroleum-based alternatives. Globally, several countries have begun to adopt differentiated bioplastics regulations and LCA standards, which directly influence the scope of algal-based materials. In the European Union, the circular economy action plan (2020) and the European Green Deal promote biodegradable and biobased plastics bags by standardised LCA practices (ISO 14040/44, EN 13432). These frameworks mandate a composability benchmark and provide funding through Horizon Europe (European Commission 2020). The EU Taxonomy Regulation (2022) now also includes algae-derived materials as sustainable investments while meeting specific carbon and land use criteria (EUBP 2023). In the United States, although the regulatory environment is less unified, the USDA BioPreferred Programme encourages the procurement of biobased products, and states like California have enacted legislation such as SB 54, which requires LCA disclosures and composability standard based on ASTM D6400 (USDA 2023; CalRecycle 2022). In China, algae-based plastics are emerging within national strategies aimed at reducing single-use plastics, guided by the 5-Year Green Plan, while LCA modeling for algae systems is being piloted in provinces like Zhejiang and Shenzhen (Zhao et al. 2023). India follows Plastic Waste Management Rules (2022), which allow certified compostable plastics under BIS/ISO standards and support algae-based R&D through the Department of Biotechnology (MoEFCC 2022; DBT 2023).

In Japan and South Korea, algal bioplastics are regulated under rigorous carbon footprint and biodegradability standards (ISO 14067, KEITI guidelines), with Japan aiming to produce 2 million tons of bioplastic annually by 2030 (METI 2021). However, inconsistencies in biodegradation definitions, labeling systems, and certification requirements across these countries pose a challenge for cross-border commercialization. Establishing internationally harmonized standards and incorporating algal bioplastics into carbon credit trading, green investment taxonomies, and circular economy indicators will be crucial to unlocking global trade and investment opportunities (Yadav and Nikalje 2024). Beyond policy, several technical and ecological research questions remain unsolved. First, optimizing algal strains through advanced genetic and metabolic engineering can significantly enhance the biosynthesis of biopolymers such as PHAs and PLA, while potentially improving the enzymatic pathway for plastic degradation. Second, innovations in bioprocess engineering, particularly the development of scalable and engineer-efficient photobioreactor systems, should focus on integrating real-world waste inputs such as carbon dioxide or nutrient-rich wastewater to align with circular economy principles. Finally, it is crucial to formulate multi-level policy instruments that combine targeted economic incentives (e.g., subsidies, tax credits), enforceable safety and quality standards, and public engagement initiatives to foster broader acceptance and deployment of algal plastic in diverse sectors. To address these challenges, future research must adopt a multidisciplinary approach. One key focus should be the optimization of algal strains through advanced genetic and metabolic engineering techniques to enhance both biopolymer yield and the enzymatic efficiency of the plastic degradation pathway. Designing robust photobioreactor systems and developing a multidisciplinary roadmap linking biotechnology, environmental science, and global governance is essential to unlocking the full potential of algae bioplastics. By embedding sustainability into both research and regulation, microalgae become central to a cleaner, circular, and biologically regenerative economy.

## Conclusion

This review highlighted the diverse potential of microalgae as a feedstock for biopolymer production, particularly PHA and PLA. Algae offers distinct advantages such as rapid growth, carbon sequestration, use of non-arable land, and compatibility with wastewater streams. These traits make them more attractive than starch or sugar-based biomass. Recent advances in metabolic and genetic engineering, including CRISPR-Cas systems, have enhanced polymer yields and carbon flux toward bioplastic pathways. Process integration through biorefineries and the adoption of biofilm-based cultivation systems are improving energy efficiency and economic feasibility. However, challenges related to the high cost of downstream processing, yield consistency, and techno-economic feasibility remain. Life cycle assessment (LCA) has become essential to evaluate sustainability claims, but standardized methodologies tailored for algal-based systems are still evolving. By integrating technological innovation with regulatory support, algal bioplastics can become a key contributor to the circular bioeconomy.

## Data Availability

Data will be made available on request.
